# Definition of the terms used to describe psoriatic disease datasets: a HIPPOCRATES initiative

**DOI:** 10.1177/1759720X261424371

**Published:** 2026-03-31

**Authors:** Oliver FitzGerald, Catherine Smith, Christine Huppertz, Martin Brom, Dario Bruno, Philip S. Helliwell, Marleen van de Sande, Maarten de Wit, Lars Werner, Vassilios Ioannidis, Diana Marek, Mark Ibberson, Stephen R. Pennington, Michaela Koehm, Frank Behrens

**Affiliations:** School of Medicine, Conway Institute for Biomolecular Research, University College Dublin, Belfield, Dublin 4, Ireland; St John’s Institute of Dermatology, King’s College London, London, UK; Translational Research, Novartis Biomedical Research, Basel, Switzerland; Translational Medicine, Novartis Biomedical Research, Basel, Switzerland; Rheumatology and Clinical Immunology, IRCCS Humanitas Research Hospital, Rozzano, Milan, Italy; Leeds Institute of Rheumatic and Musculoskeletal Medicine, University of Leeds, Leeds, UK; Department of Rheumatology and Clinical Immunology, Amsterdam Institute for Immunology and Infectious Diseases, Amsterdam UMC, University of Amsterdam, Amsterdam, The Netherlands; Amsterdam Rheumatology and Immunology Center, Amsterdam, The Netherlands; HIPPOCRATES Patient Research Partner, Amsterdam, The Netherlands; HIPPOCRATES Patient Research Partner, Copenhagen, Denmark; Vital-IT Group, SIB Swiss Institute of Bioinformatics, Lausanne, Switzerland; Vital-IT Group, SIB Swiss Institute of Bioinformatics, Lausanne, Switzerland; Vital-IT Group, SIB Swiss Institute of Bioinformatics, Lausanne, Switzerland; Conway Institute for Biomolecular Research, School of Medicine, University College Dublin, Dublin, Ireland; Translational Rheumatology, Immunology – Inflammation Medicine, University Medicine Goethe-University, Fraunhofer Institute for Translational Medicine and Pharmacology ITMP and Fraunhofer Cluster of Excellence Immune Mediated Diseases, Frankfurt, Germany; Translational Rheumatology, Immunology – Inflammation Medicine, University Medicine Goethe-University, Fraunhofer Institute for Translational Medicine and Pharmacology ITMP and Fraunhofer Cluster of Excellence Immune Mediated Diseases, Frankfurt, Germany

**Keywords:** dataset terms, psoriasis, psoriatic arthritis

## Abstract

Psoriatic arthritis (PsA) is a complex, multisystem disease with no diagnostic criteria and discordant approaches to assessing disease activity and response to treatment. PsA is part of the Psoriatic Disease spectrum, encompassing cutaneous psoriasis and nail changes, PsA and in some cases, uveitis and inflammatory bowel disease. The HIPPOCRATES consortium is addressing unmet needs in PsA and has now established a harmonised data platform, the Secure HIPPOCRATES Data Management Platform (SHDMP), which will be transformative in providing researchers with the ability to interrogate at scale psoriatic disease datasets. The SHDMP datasets are diverse and include people with psoriasis being followed for the development of PsA, as well as cross-sectional, observational and randomised control trial data on people with PsA. To better understand and describe the nature of the SHDMP datasets, the HIPPOCRATES consortium has now defined and agreed on the terms that will be applied to each dataset. In this short report, the process adopted to obtain consensus on the dataset definition and the final set of terms agreed upon is described and discussed. The agreement on the terms to be used will greatly enhance a researcher’s ability to select the appropriate SHDMP datasets to study.

## Plain language summary

How best to describe psoriatic diseases datasets? A HIPPOCRATES initiative to describe the terms that are best used

Psoriatic arthritis (PsA) is a complex disease which is difficult to diagnose and which responds variably to treatment. The HIPPOCRATES consortium is addressing unmet needs in PsA and has now established a large clinical data platform, the Secure HIPPOCRATES Data Management Platform (SHDMP). The SHDMP datasets are quite varied and include people with psoriasis being followed for the development of PsA, as well as cross-sectional, observational and randomised control trial data on people with PsA. To better understand and describe the nature of the SHDMP datasets, the HIPPOCRATES consortium has now defined and agreed the terms which will be applied to each dataset. In this short report, the process adopted to obtain consensus on dataset definition and the final set of terms agreed is described and discussed. The agreement on the terms to be used will greatly enhance a researcher’s ability to select the appropriate SHDMP datasets to study.

## Introduction

HIPPOCRATES (https://www.hippocrates-imi.eu/) is a large European public–private partnership funded by the Innovative Medicines Initiative to address some of the major unmet needs in Psoriatic Arthritis (PsA).^1^ One of the key strengths within HIPPOCRATES is the access provided by both academic and industry partners to up to 30 datasets of patients with PsA or psoriasis patients being prospectively followed for the development of PsA. A centralised database, the Secure HIPPOCRATES Data Management Platform (SHDMP), has been created at the Swiss Institute for Bioinformatics, where both pseudonymised and harmonised clinical and molecular data can be stored. The molecular data being uploaded will in some datasets include data on genomics, epigenetics, targeted and unbiased proteomics, metabolomics and lipidomics. To date, data from 16 prioritised datasets and >7000 patients have been uploaded to the platform. The SHDMP now represents the largest repository of psoriatic disease clinical and molecular data in Europe.

Pending new approval from respective consortium members, it is the intention of HIPPOCRATES to make the SHDMP data available to both consortium partners as well as external parties post-HIPPOCRATES in 2026/27. This rich dataset collection will be of considerable value to those involved in psoriasis and PsA research, in particular, where there is a need to validate findings in independent datasets. Given the complexity and heterogeneity of the datasets, it has become apparent that a short, searchable term for each dataset type is required to assist those unfamiliar with the datasets and those without a clinical background in identifying datasets of interest. For example, a rheumatologist interested in validating biomarkers associated with radiographic joint damage will want to identify datasets where clinical (disease activity) and radiographic data have been obtained both at baseline and after a period of follow-up. Having a term that facilitates such identification will greatly assist in this task.

In this study, we report the development of a set of terms that can readily be applied to PsA datasets and to datasets of psoriasis patients being prospectively followed for the development of PsA. These terms were initially proposed and discussed at the 2024 annual plenary meeting of HIPPOCRATES, subsequently modified and following two rounds of online surveys (SurveyMonkey, www.surveymonkey.com), approved for use by the HIPPOCRATES consortium. This approach is in line with standard approaches to achieving consensus.^2^ It is proposed that these terms will also be useful to other external parties interested in psoriatic disease research who are seeking to compare or to validate their initial findings.

## Methods

Following initial discussion between experienced HIPPOCRATES clinicians, proposals were presented and discussed at the 2024 annual plenary meeting, where all members of the HIPPOCRATES interdisciplinary consortium, including clinicians, basic scientists, industry partners and Patient Research Partners (PRPs), had an opportunity to comment and discuss the first version (V1) of proposed terms to be used to describe the datasets. Comments were incorporated into an updated version (V2) of the proposed terms and circulated to all HIPPOCRATES members using an online survey tool. Binary approval for V2 was sought and further comments solicited from all HIPPOCRATES members, who were first asked to declare if they considered themselves qualified enough to provide comments. Based on the comments received from those who felt qualified to respond, V2 was further refined into a new version (V3) and again submitted for voting using the same online tool. The proposal would be considered approved if a threshold of at least 80% of positive votes was received; otherwise, the process was to be repeated, with further refinement of the proposed terms, until a consensus was met. While Outcome Measures in Rheumatology Clinical Trials (OMERACT) previously proposed a 70% consensus threshold,^3^ we chose to use a more stringent 80% threshold as proposed by Humphrey-Murto et al.^4^

## Results

All HIPPOCRATES clinical (*n* = 35), scientific (*n* = 35), industry (*n* = 30) and PRP (*n* = 15) members were asked to complete the survey if they felt sufficiently qualified to do so. Of the 29 responses received, 8 did not feel qualified to fill out the survey. Of the 21 respondents who felt qualified, there were 12 clinicians (34%), 3 PRPs (20%), 3 industry partners (10%) and 3 basic scientists (8.6%). V2 received 71% approval, and 6 further comments were received. These comments were reviewed and incorporated where appropriate into a new version (V3), which was circulated again for approval (Table 1). A 94% approval rating was obtained.

**Table 1.** Terms to be used for psoriasis and psoriatic arthritis datasets.

Term	Definition	Clarification
Pso	People with Pso where they have not been formally assessed as to whether they have any MSK involvement	
PsC	PsC where there is no clinical evidence of MSK involvement	
PsC-Ineg	PsC without clinical or imaging evidence of MSK involvement	
Pso-MSK	People with Pso who have MSK symptoms (e.g. arthralgia, back pain or stiffness) or imaging findings to suggest MSK involvement but who do not meet CASPAR criteria for PsA	
PsA	PsA, not formally assessed for CASPAR criteria; not assessed for MSK DA or SD on imaging	
PsA-Casp	PsA in accordance with CASPAR criteria; not assessed for MSK DA or SD	
	-DA	where patients have been assessed for MSK DA
	-SD	where patients have been assessed for SD
Treatment	Definition	
PsC, Pso or PsA	-naïve	On symptomatic treatment only^a^
	-cs	On csDMARD
	-ts	On tsDMARD

Features, such as diseases and treatments, can be mixed; for example PsA-Casp-DA-SD-ts is a dataset of people with PsA, meeting CASPAR, with disease activity and structural damage assessed and is undergoing treatment with a tsDMARD. (SD assessment by some imaging techniques (e.g. ultrasound) may also detect inflammatory change.)

a Symptomatic treatment includes topical treatment, UVB, NSAIDs and corticosteroids.

CASPAR, ClASsification for Psoriatic Arthritis; csDMARD, conventional synthetic disease-modifying anti-rheumatic drug; DA, disease activity; MSK, musculoskeletal; PsA, psoriatic arthritis; PsC, cutaneous psoriasis; Pso, psoriasis; SD, structural damage; tsDMARD, targeted (small molecule or biologic) synthetic disease-modifying anti-rheumatic drug.

## Discussion

Following discussion both in-person and online, a 94% approval rating was obtained for a set of terms to describe the datasets included on the SHDMP. The low response rate to the survey reflects the composition of the HIPPOCRATES consortium, with most members deeming themselves not qualified to address the clinical issues raised. While the overall response rate was low (21/115), more than one-third of clinician members, including the only Dermatologist partner, responded to the survey and were actively involved in the discussion process. The agreed terms have now been applied to 16 datasets, facilitating the easy identification of datasets of interest. This will be of benefit to HIPPOCRATES members, but following completion of the HIPPOCRATES project, will also potentially benefit external parties accessing the platform.

The terms apply predominantly to PsA datasets, although datasets of psoriasis patients being followed for the development of PsA are also included in the SHDMP. Such datasets are the particular focus of HIPPOCRATES research efforts. For datasets with PsA, those exploring the SHDMP will be able to quickly ascertain whether CASPAR criteria were used to select patients, whether disease activity or structural damage was assessed and whether the dataset of patients was treated with any conventional or targeted synthetic treatments, as in a randomised controlled trial or long-term observational study. The terms will also be useful for datasets of psoriasis patients being followed for the development of PsA and they could be used to define psoriasis datasets on symptomatic, conventional or targeted therapies.

As the HIPPOCRATES project enters its final period, sustainability is under active consideration. Undoubtedly, future projects will wish to include access to the SHDMP, using the terms which have been agreed and implemented to identify relevant cohorts. Some of these projects will include current HIPPOCRATES partners but it is likely that requests to access the data may also arise from external groups. Such access will be reviewed by the data access committee, using a data access policy developed and approved by the HIPPOCRATES consortium. It is also considered likely that external researchers working with other datasets will want to apply the same terminology to their datasets so as to facilitate comparison and validation of findings.

### Study limitations

It is acknowledged that the terminology used may need to evolve over time as additional datasets are uploaded to the SHDMP. During the lifetime of the HIPPOCRATES consortium, any required changes, such as the addition of PsA or psoriasis subtypes, will be considered and approved by the consortium. To address any required changes post-HIPPOCRATES, a multi-stakeholder data access committee has been established to provide structured governance to the SHDMP, including any requirement to update the terminology. Finally, it is also acknowledged that the number of respondents to the online surveys is low, but it is representative of the consortium as a whole and the respondents included both people with PsA as well as many with a long-term, specialist interest in psoriatic disease.

### Summary

This study describes the process adopted to agree on a set of terms to define the datasets uploaded to the largest European data management platform for people with psoriatic disease. The final agreed set of terms is also defined. This set of terms will be of benefit in the future to all investigators, including external investigators, who are interested in psoriatic disease research.

## Declarations

**Ethics approval and consent to participate:** Not applicable.

**Consent for publication:** All authors provided consent for publication.

**Availability of data and materials:** Not applicable.
